# Nanoencapsulation of Eggplant (*Solanum melongena* L.) Peel Extract in Electrospun Gelatin Nanofiber: Preparation, Characterization, and In Vitro Release

**DOI:** 10.3390/nano12132303

**Published:** 2022-07-04

**Authors:** Danya Elizabeth Estrella-Osuna, José Agustín Tapia-Hernández, Saúl Ruíz-Cruz, Enrique Márquez-Ríos, José de Jesús Ornelas-Paz, Carmen Lizette Del-Toro-Sánchez, Víctor Manuel Ocaño-Higuera, Francisco Rodríguez-Félix, María Isabel Estrada-Alvarado, Luis Alberto Cira-Chávez

**Affiliations:** 1 Departamento de Biotecnología y Ciencias Alimentarias, Instituto Tecnológico de Sonora, 5 de Febrero 818 sur, Ciudad Obregón 85000, Sonora, Mexico; daniaestrella_@hotmail.com (D.E.E.-O.); mestrada@itson.edu.mx (M.I.E.-A.); luis.cira@itson.edu.mx (L.A.C.-C.); 2 Departamento de Investigación y Posgrado en Alimentos, Universidad de Sonora, Encinas y Rosales s/n, Hermosillo 83000, Sonora, Mexico; enrique.marquez@unison.mx (E.M.-R.); carmen.deltoro@unison.mx (C.L.D.-T.-S.); francisco.rodriguezfelix@unison.mx (F.R.-F.); 3 Centro de Investigación en Alimentación y Desarrollo, Av. Río Conchos s/n, Parque Industrial, Cuauhtémoc 31570, Chihuahua, Mexico; jornelas@ciad.mx; 4 Departamento de Ciencias Químico Biológicas, Universidad de Sonora, Encinas y Rosales s/n, Hermosillo 83000, Sonora, Mexico; victor.ocano@unison.mx

**Keywords:** eggplant peel, gelatin, electrospun, nanofiber, in vitro release

## Abstract

This study describes the preparation and characterization of eggplant peel extract-loaded electrospun gelatin nanofiber and study of its in vitro release. Results obtained by scanning electron microscopy (SEM) and transmission electronic microscopy (TEM) micrograph revealed that eggplant peel extract-loaded electrospun gelatin nanofiber is in nanometric range with an average diameter 606.7 ± 184.5 and 643.6 ± 186.7 nm for 20 and 33.3 mg mL^−1^ of extract addition, respectively. Moreover, the incorporation of extract improved morphology by being smooth, homogeneous, and without account formation compared to nanofibers without extract (control). Fourier transform-infrared (FT-IR) spectra indicated that interaction exists between electrospun gelatin nanofiber and eggplant peel extract by hydrogen bond interactions, mainly. Electrospun gelatin nanofibers showed encapsulation efficiency greater than 90% of extract and a maximum release of 95 and 80% for the medium at pH 1.5 and 7.5, respectively. Therefore, the electrospinning technique is a good alternative for the conservation of bioactive compounds present in the eggplant peel through electrospun gelatin nanofiber.

## 1. Introduction

Currently, the food industry generates a large amount of waste and by-product considered inedible, mainly of plant tissue parts such as peel, seed, husk, and oil cake, among others [1,2,3,4]. Eggplant (*Solanum melongena* L.) is a crop of non-tuberous species of nightshade family (*Solanaceae*) that generates a large amount of these by-products [5]. This crop is considered agronomically and economically important [6], and the fruit is present in the diet of many countries, especially India and Bangladesh, southeast Asia, and the Middle East [7]. Eggplant cultivation amounts to around 50 million tons, produced on more than 1,800,000 ha worldwide [6]. Specifically, eggplant peel as a by-product is an important source of different bioactive compounds that can be recovered, such as anthocyanins, especially delphinidin 3-rutinoside (tulipanin) and delphinidin 3-(p-coumaroyl rutinoside)-5-glucoside (nasunin), and of other polyphenols such as 5-O-caffeoylquinic acid (chlorogenic acid) [5,8].

Recent studies show that the consumption of eggplant contributes to the decrease of the appearance of chronic degenerative diseases, due to the bioactive compounds present in the peel [9]. In addition, the antioxidant activity is the main biological activity that is conferred to the eggplant peel extract [10,11]. Todaro [12] extracted anthocyanins from eggplant peel, evaluating three extraction solvents and antioxidant activity. Results showed that tartaric acid was more efficient than malic acid in extraction yield, and similar ethanol acidified. Furthermore, delphinidin-3-rutinoside was extracted and identified as the major compounds and antioxidant activity was higher in malic acid. Di Sotto [13] evaluated antioxidant activity of two extracts named DR2B and DR2C. Results showed that chlorogenic acid and delphinidin-3-rutinoside were the major constituents and antioxidant activity by DPPH and ABTS was higher in extract DR2B that in extract DR2C.

However, the biological activity of bioactive compounds could be affected by chemical, physical, and physiological aspects such as pH, O_2_, temperature, and enzymatic activity affecting its potential therapeutic activity [14]. Moreover, a potential health risk is undertaken due to chemical instability and high degradability when supplied orally and during its path through the stomach due to its low pH [15]. An alternative to the harmful effects is the nanoencapsulation of extracts using matrices that help protect these compounds [16,17]. The importance of applying nanotechnology in nutraceutical and bioactive compounds is its protection against changes in pH during its incorporation into a food, improving its bioavailability when passing through the gastrointestinal tract and not losing its biological activity. In addition, the current regulations of each country for its nanoformulation and incorporation into biological systems is worth noting [18].

**Figure 1:**
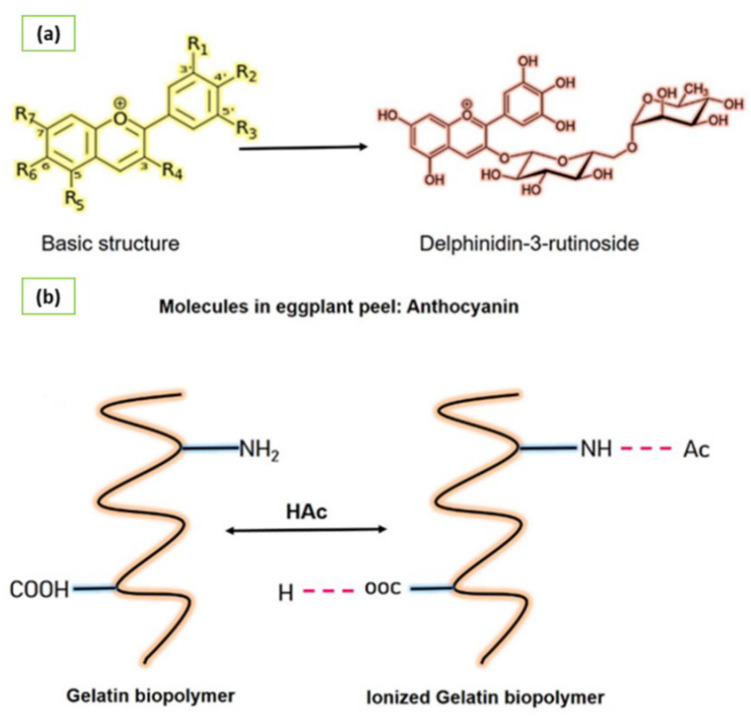
Raw materials for the formation of gelatin nanofiber by electrospinning. (**a**) Main molecules present in eggplant (*Solanum melongena* L.) peel extract and (**b**) Behavior of gelatin polymer in acetic acid-water (30% *v*/*v*) as a solvent for the formation of nanofibers.

Biopolymers have been used for nanoencapsulation of extracts to be recognized as generally recognized as safe (GRAS) materials [19,20] for food and pharmaceutical applications. Some studies have encapsulated extracts in biopolymers such as yerba mate extract in alginate and chitosan [21], tomato extract in gelatin [22], and elderberry extract in phospholipid [23], among others. Mainly, gelatin has been widely used for nanoencapsulation because it is a biopolymer obtained by denaturing collagen. Other characteristics are availability, biocompatibility, biodegradability, and non-immunogenicity, especially in biological applications [24]. Figure 1 shows the raw materials for obtaining of gelatin nanofiber by electrospinning.

In this sense, different methods have been proposed to nanoencapsulate extracts in biopolymers such as electrohydrodynamic atomization, nano spray dryer, antisolvent-dialysis [25], and micro/nanofluidics [26,27]. This is thanks to the fact that the food industry together with nutraceuticals is opening the way to reveal exclusive properties and high surface/volume ratio thanks to its small size, being able to guarantee purer compounds and a better application of them [28], specifically, electrohydrodynamic atomization (EHDA) processes including electrospraying and electrospinning techniques [20,29] for the formation of nanoparticles and nanofibers, respectively. Electrospinning is a suitable method due to its ability to simply produce nanofibers using a large variety of materials [30] such as biopolymers. Nanofiber formation is based on three stages, (1) onset of jetting and rectilinear jet development; (2) bending deformation with looping and spiraling trajectories, and nanofiber solidification with evaporation of solvents; (3) nanofibers collection [31]. Furthermore, electrospun nanofibers have numerous advantages such as high surface area to volume ratio, nanometric scale, and porous structure, providing unique properties to the system [32].

Therefore, the present study includes the encapsulation of eggplant (*Solanum melongena* L.) peel extract in electrospun gelatin nanofibers as an in vitro release system. Nanofibers were characterized by SEM and TEM to observe the morphology, fiber size distribution, average diameter, and polydispersity index. In addition, FT-IR was carried out to observe the possible interactions between gelatin and eggplant skin extract; as well as encapsulation efficiency and in vitro release of the compounds present in the material at two pH (1.5 and 7.5).

## 2. Materials and Methods

### 2.1. Chemical Reagents

Gelatin (powder food grade, Merck, 104078) was purchased from Merck (Burlington, MA, USA). Ethanol and acetic acid were purchased from Fagalab (Sinaloa, Mexico). Distilled water was also used.

### 2.2. Preparation of Eggplant Peel Extract

Firstly, eggplants were purchased at a local store and subsequently transferred to the Emerging Technologies Laboratory of the Center for Biotechnology, Agricultural, and Environmental Research and Innovation (CIIBAA) of Technological Institute of Sonora (ITSON) in Cd. Obregón Sonora, Mexico. A representative sample of the lot was taken, and the pulp was separated from the peel. Then, the peel was cut into slices 1 cm thick.

For the obtaining of extract, the methodology described by Stoll [33] was used. First, 3 g of samples was homogenized in 30 mL of a 70% (*v*/*v*) ethanol solution, acidified with 1% of HCl and at pH of 3.5 in a ratio (1:10 *p*/*v*). It was left for 1 h under constant stirring at 35 °C in an IKA C-MAG HS7 homogenizer. Subsequently, vacuum filtration was performed, recovering the supernatant and stored in the dark until use.

### 2.3. Preparation of Solutions

Gelatin solutions containing eggplant peel extract in acidified ethanol were prepared from the methodology proposed by Hani [34]. Firstly, 40% *w*/*v* gelatin solutions were prepared in 30% *v*/*v* acetic acid solutions with two amounts of extract 5 and 3 mL which correspond to concentrations of 33.3 and 20 mg mL^−1^, respectively, adding them to the prepared control gelatin solution. All solutions were homogenized using magnetic stirring for 1 h at 25 °C.

### 2.4. Electrospinning Process

**Figure 2:**
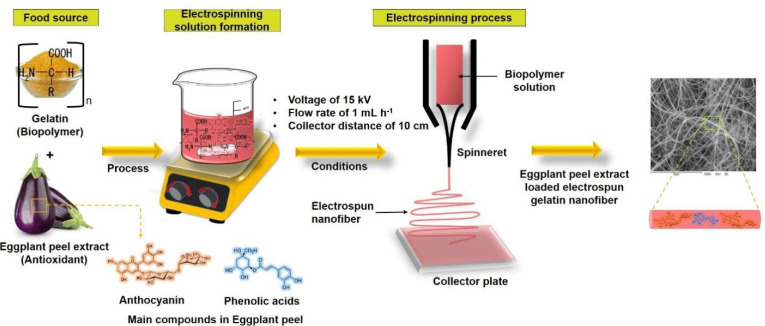
Schematic representation of electrospraying process for obtaining eggplant peel extract-loaded electrospun gelatin nanofiber.

First, 3 mL of control gelatin and eggplant peel extract-gelatin solutions were transferred into a plastic syringe with a needle of 0.8 mm diameter. Two concentrations of extract were used, 33.3 and 20 mg mL^−1^. Then, the syringe was set in a pump (KD Scientific, Holliston, MA, USA) to regulate the flow of the polymer solution. The voltage was applied using a high-voltage power source (model CZE 1000R, Spellman, Hauppauge, NY, USA). A 10 cm × 10 cm aluminum plate was utilized for electrospun nanofiber collection. The gelatin and eggplant peel extract-gelatin solutions were obtained at a voltage of 15 kV, flow rate of 1 mL h^−1^, and collector distance of 10 cm. Figure 2 shows the schematic representation of the obtaining of eggplant peel extract-loaded electrospun gelatin nanofiber.

### 2.5. Characterization of Electrospun Nanofiber

#### 2.5.1. Scanning Electron Microscopy (SEM)

The study of the morphology of electrospun gelatin nanofiber and eggplant peel extract-loaded electrospun gelatin nanofiber was by SEM using a JEOL Model JSM-7800F equipment (JEOL, Pleasanton, CA, USA). Powder of electrospun nanofiber was prepared through its immobilization on carbon-coated 400-mesh copper grids (Ted Pella, Inc., Redding, CA, USA). An acceleration voltage of 10 kV was used. Additionally, eggplant peel extract-loaded electrospun gelatin nanofibers were analyzed in particle-size distribution (PSD) and average diameter employing the ImageJ software program (NIH, Bethesda, MD, USA). Moreover, the polydispersity index (*PDI*) of electrospun nanofiber was obtained from following Equation (1):

(1) $$PDI=\frac{\sigma }{\bar{X}}\;\;$$

where *σ* represents the standard deviation and $\bar{X}$ represents the average diameter of the nanoparticles. *PDI* closer to 0 represents monodispersed particles and *PDI* close to 1 represents polydispersed particles.

#### 2.5.2. Transmission Electronic Microscopy (TEM)

The shape of eggplant peel extract-loaded electrospun gelatin nanofiber was studied by transmission electron microscopy using a JEOL equipment (JEOL, Ltd., Tokyo, Japan) at 200 kV operating voltage and a field emission filament. The preparation of the sample consisted of placing a small quantity of nanofiber sample on a 100-mesh grid, then, another 100-mesh grid was placed on top of the nanofibers and observed in a TEM equipment.

#### 2.5.3. Fourier Transform-Infrared (FT-IR) Spectroscopy

To observe the physical interaction between gelatin and eggplant peel extract, FT-IR spectroscopy was employed. A Spectrum GX FT-IR (Perkin-Elmer, Waltham, MA, USA) Infrared Spectrometer equipment was used and spectrum scans were performed in the range 4000 to 500 cm^−1^. The method was attenuated total reflectance (ATR). Measurements were performed in transmittance mode. Samples were run in triplicate.

### 2.6. Encapsulation Efficiency (EE)

The EE was carried out for eggplant peel extract-loaded electrospun gelatin nanofibers. Firstly, 0.05 g of material was added to 5 mL of 30% (*v*/*v*) acetic acid, followed by sonication (Branson 3210) for 15 min. Subsequently, the dissolved material was centrifuged (HERMLE Z 323 K) at 2340× *g* for 10 min and the supernatants were obtained. For the quantification of the concentration, tests of the total phenolic content were carried out using the Folin–Ciocalteu method, following the methodology of Hani [33] with modifications. Results of the quantification of total phenols were obtained and the efficiency was calculated according to Equation (2):

(2) $$EE\;\left(\%\right)=\frac{\left(C1-C2\right)}{C1}\times 100$$

where C1 is the initial encapsulated concentration and C2 is the final encapsulated concentration.

### 2.7. In Vitro Release

The in vitro release study was performed based on the methodology proposed by Aceval [35] with modifications. Two pH media were used for in vitro release: (1) 1 M sodium citrate solution for pH 7.5 and (2) stomach fluid simulation for pH 1.5. For the release, 0.1 g of eggplant peel extract-loaded electrospun gelatin nanofibers was weighed and suspended in 10 mL of the release medium, then kept under constant stirring at 130 rpm for 6 h. Finally, an aliquot of the supernatant was taken every hour. The analysis of total phenols was performed for each time measured according to the methodology described by Del-Toro-Sánchez et al. [36]. The reaction consisted of adding in a microplate 150 µL of Folin–Ciocalteau reagent, 30 µL of the extract, and 120 µL of Na_2_CO_3_. The reaction was left to stand for 30 min in complete darkness and the absorbance reading was taken at 750 nm in the microplate reader (Thermo Scientific Multiskan Sky, Vantaa, Finland). The percentage of release was calculated from the determination of phenolic compounds present, substituted in Equation (3):

(3) $$\mathrm{phenolic}\;\mathrm{release}\left(\%\right)=\frac{Mt}{M\infty }\times 100$$

where *Mt*/*M∞* is the fraction of phenols mass released at time t  with respect to the maximum mass of phenols that would be released at time t=∞.

### 2.8. Statistic Analysis

Descriptive statistic of means and standard deviation (SD) were used for all analyses. For comparison of means of the encapsulation efficiency, the Tukey test was employed at a 95% confidence level (*p <* 0.05) using Infostat 2008 software.

## 3. Results and Discussion

### 3.1. Morphology of Eggplant Peel Extract Loaded Electrospun Gelatin Nanofibers

#### 3.1.1. Morphology by SEM

**Figure 3:**
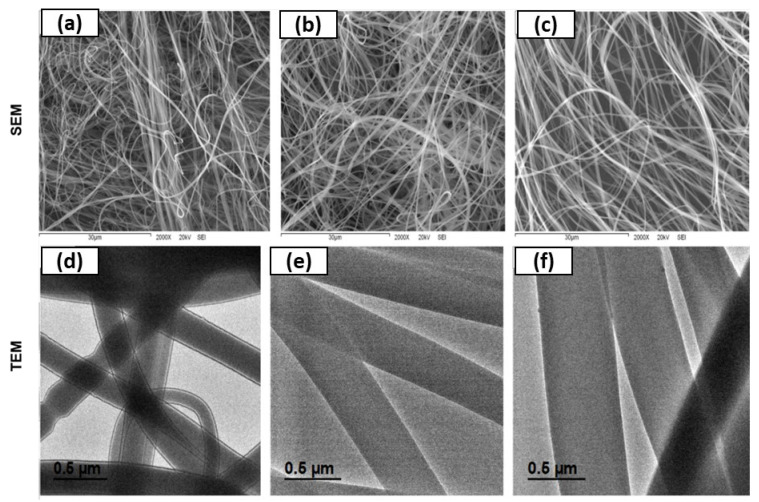
Micrographs by SEM and TEM of eggplant peel extract-loaded gelatin electrospun nanofiber. (**a**,**d**) electrospun gelatin nanofiber, (**b**,**e**) nanofiber loaded with 33.3% eggplant peel extract, and (**c**,**f**) nanofiber loaded with 20% eggplant peel extract.

Figure 3 shows the micrographs by SEM of electrospun gelatin nanofibers obtained with 40% (*w*/*v*) gelatin and two concentrations of eggplant peel extract, 33.3 and 20 mg mL^−1^. Firstly, electrospun gelatin nanofiber was prepared to the following equipment conditions of voltage 15 kV, flow rate of 1 mL h^−1^, and distance of the collector to the needle of 10 cm. Control nanofibers at 40% (*w*/*v*) showed morphology of elongated fibers with jet chain entanglement (Figure 3a). This concentration favored the formation of the nanofiber without beads. The same behavior was observed in eggplant peel extract-loaded electrospun gelatin nanofibers, where the extract concentrations used in this study were not a factor for bead formation (Figure 3b,c).

Another study by Hani [34] reported obtaining gelatin nanofibers by electrospinning method at different gelatin concentration. Results of Hani were similar to the current study where with gelatin concentration of 40% (*w*/*v*), good electrospun nanofibers were formed. Moreover, at a lower concentration, there was bead formation mainly due to surface tension dominance over viscoelastic forces resulting in the absence of chain entanglement between polymer molecules. Furthermore, once the extract was nanoencapsulated in electrospun nanofiber, they obtained fibers without beads. This was similar in the current study for both concentrations of added eggplant peel extract.

#### 3.1.2. Morphology by TEM

In addition to the study of morphology by SEM, it was also studied by TEM (Figure 3). This technique showed that electrospun nanofibers obtained only with gelatin and compared with eggplant peel extract and gelatin showed differences in thickness, observing a significant increase in size. Moreover, morphology with wave formations in some nanofibers were observed in unencapsulated electrospun nanofiber (Figure 3a), and not detected by the SEM technique. However, when the extract was added, waves were not observed in electrospun nanofibers (Figure 3b,c). This concludes that the addition of extract to the electrospun nanofiber improves its contour, obtaining smooth fibers. Furthermore, the fiber diameter in nanometer range was confirmed by this technique.

### 3.2. Fiber Size Distribution

**Figure 4:**
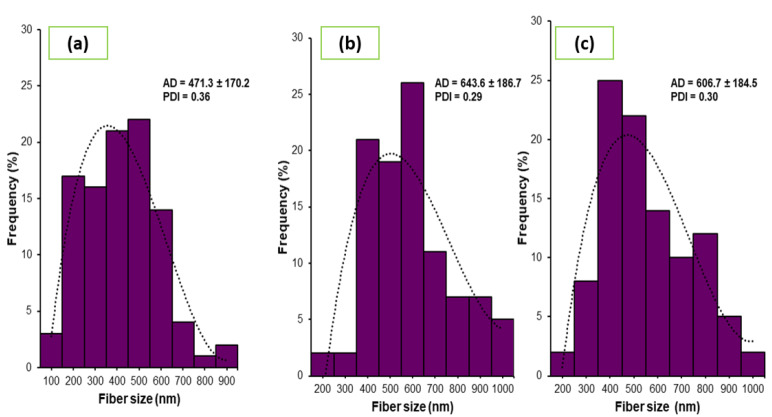
Particle size distribution, average diameter, and polydispersity index of eggplant peel extract-loaded electrospun gelatin nanofiber. (**a**) Electrospun gelatin nanofiber, (**b**) nanofiber loaded with 33.3% eggplant peel extract, and (**c**) nanofiber loaded with 20% of eggplant peel extract.

Figure 4 shows the fiber size distribution, average diameter (AD), and polydispersity index (*PDI*) of controlled electrospun gelatin nanofibers and eggplant peel extract-loaded electrospun gelatin nanofibers. For control nanofibers, the average diameter of the fiber size was of 471.3 nm. However, a significant increase was observed when the extract was added, in both concentrations. First, for the concentration of 33.3 mg mL^−1^ of eggplant peel extract added, fiber size increased 36.5% with respect to the control electrospun nanofibers, obtaining an average diameter of 643.6 ± 186.7 nm. On the other hand, when 20 mg mL^−1^ of eggplant peel extract was added, fiber size increased 28.7%, respect to the control electrospun nanofibers obtaining an average diameter of 606.7 ± 184.5 nm. However, from the extract with a concentration of 20 mg mL^−1^ to the extract of 33.3 mg mL^−1^, there was an increase of 5.7%. This means that the higher the concentration of the nanoencapsulated extract, the larger the diameter of the fiber obtained. Vafania [30] mentioned that the increase in the diameter in the fibers after being loaded with extracts can be attributed to the reduction of the electrical conductivity; therefore, a low conductivity value produces a reduction in the elongation of the polymeric jet within the electric field. The same behavior was detected in a study by Amjadi [37], where the diameter of processed fibers increased according to the concentration of gelatin, as well as its viscosity, determining that this parameter is the most important for determining the morphology of the electrospun fiber. Tavassoli-Kafrani [38] prepared fibers to encapsulate phenolic compounds. They also obtained greater diameters in the extract-loaded gelatin nanofibers, compared to control gelatin nanofibers (gelatin), attributing this result to the molecular size of the encapsulated compounds, which may be the result of the fiber increase elaborated in this study.

Control electrospun gelatin nanofibers (Figure 4a) showed distribution from 182.7 to 984.1 nm; however, 42% of the size of the electrospun nanofiber appeared between 408.6 and 589.3 nm. On the other hand, the electrospun nanofiber loaded with 33.3 mg mL^−1^ of eggplant peel extract (Figure 4b) showed a fiber size distribution from 231.2 to 1111.6 nm, with highest accumulation of fiber size from 408.6 to 698.3 nm, corresponding to 66% of total sizes. In addition, the concentration of 20 mg/mL of eggplant peel extract incorporated in electrospun gelatin nanofiber (Figure 4c) showed a fiber size distribution from 231.1 to 1119.7 nm, and similar to the concentration of 33.3 mg mL^−1^, this concentration showed a high accumulation in fiber size from 410.3 to 687.1 nm with 61% of the fiber size.

Figure 4 shows the *PDI* of electrospun gelatin nanofiber loaded with two concentrations of extract, 33.3 and 20 mg mL^−1^. These data were obtained by dividing the standard deviation of the nanoparticles and the average diameter. Firstly, for nanofibers without extract (control), the *PDI* was of 0.36; however, once the extract was incorporated, the *PDI* were 0.29 and 0.30 for 33.3 and 20 mg mL^−1^, respectively. These results show that the addition of extract decreases the *PDI* of the electrospun gelatin nanofiber compared to control *PDI*, producing fiber with narrow size distribution. Danaei [39] considered that values of *PDI* under of 0.2 are commonly acceptable in nanoparticles that are created from polymers and are considered monodispersed particles. Therefore, according to the results, nanofibers loaded with the two concentrations of eggplant peel extract have a tendency to be monodispersed, since they are very close to 0.2 compared to the control electrospun gelatin nanofiber.

### 3.3. Interaction of Eggplant Peel Extract-Loaded Electrospun Gelatin Nanofiber

**Figure 5:**
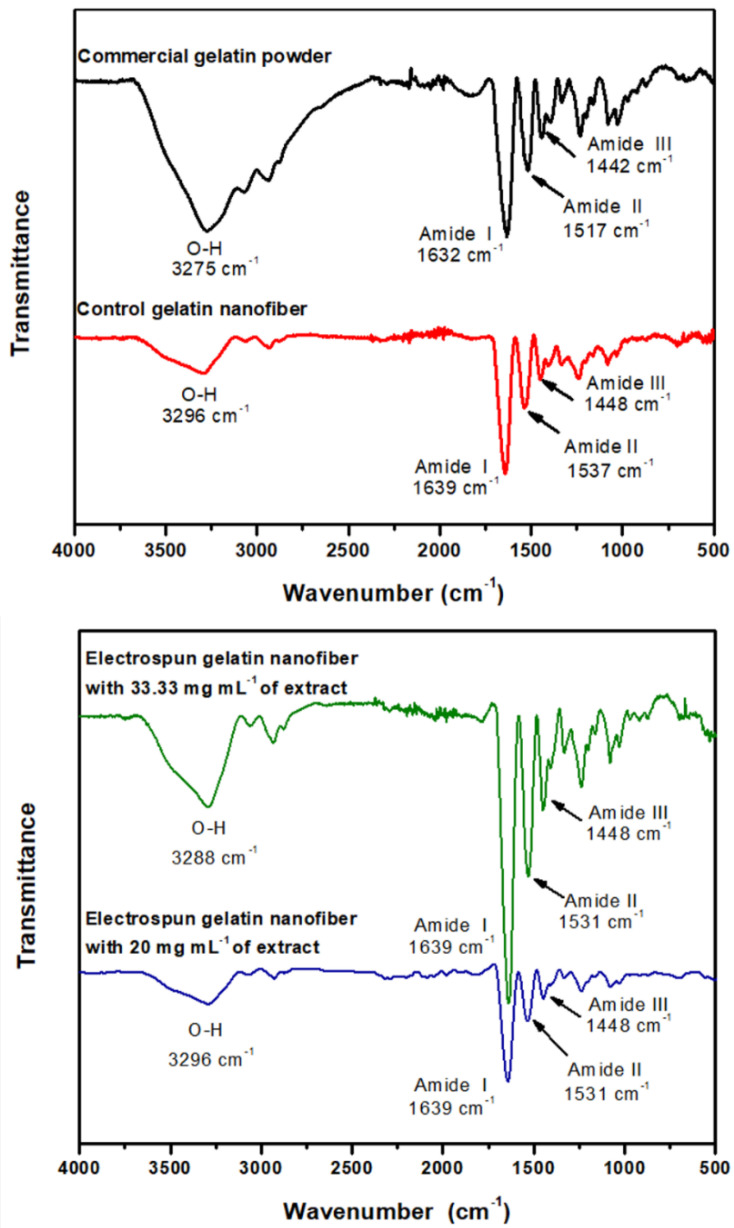
Infrared spectra of gelatin powder, control gelatin nanofiber, and electrospun gelatin nanofiber with 33.3 and 20% of eggplant peel extract.

In this analysis, infrared spectroscopy was used for the identification of functional groups. The FT-IR has proven to be useful for more advanced studies aimed at the characterization of polymeric and biopolymeric materials. The spectra of gelatin and electrospun nanofibers with two extract concentrations observed in Figure 5. Commercial gelatin powder showed the characteristic bands of a protein. Firstly, amide band I corresponds to the vibratory stretching of −C=O at 1632 cm^−1^, amide band II corresponds to the vibration of flexion of the −NH bond and the vibration of the CN at 1517 cm^−1^, amide band III corresponds to a complex mixture of displacement at 1442 cm^−1^. Further, the band of −OH and −NH corresponds to the vibrational stretch bond at 3275 cm^−1^ and the band around of 2900 cm^−1^ represents a stretching of the C−H bond. The infrared spectrum for electrospun gelatin nanofiber (control) showed four characteristic bands that were exhibited in commercial gelatin powder, but with significant shifts, amide band I at 1661 cm^−1^, amide band II at 1537 cm^−1^, amide band III at 1455 cm^−1^, and the −OH band and −NH at 3321 cm^−1^. These differences are mainly due to the conformational rearrangement of gelatin when it passes from raw material into an electrospun nanofiber form, due to the interaction with the solvent and electrospinning conditions used, increasing hydrogen-bonding interactions, mainly intramolecular hydrogen bonding.

The infrared spectrum of electrospun nanofiber with a concentration of 33 mg mL^−1^ of extract showed the amide band I at 1639 cm^−1^, amide band II at 1531 cm^−1^, amide band III at 1448 cm^−1^, and the band of −OH and −NH at 3288 cm^−1^. Furthermore, the bands at 1600, 1372, and 1258 cm^−1^ are attributed to characteristic vibrations of the aromatic group of the anthocyanins and other phenolic compounds present in the extract. In this sense, the main change was observed in the −OH and −NH band, where there was an increase in the width of band compared to the electrospun gelatin nanofiber control. This increase may be due to the appearance of −OH groups present in phenolic compounds, with anthocyanins as the major molecules present. However, the shifting of the −OH and −NH band from 3296 to 3288 cm^−1^, from control nanofiber to eggplant peel extract-loaded electrospun gelatin nanofibers, shows the presence of hydrogen bonds interactions of the −NH groups of the protein with the −OH groups present in the extract, mainly intermolecular hydrogen bonding. On the other hand, infrared spectrum of electrospun nanofiber with a concentration of 20 mg mL^−1^ of extract showed the amide band I at 1639 cm^−1^, amide band II at 1531 cm^−1^, amide band III at 1448 cm^−1^, and the band of −OH and −NH at 3296 cm^−1^. In this spectrum, no changes were observed in the amide band I, II, and III with respect to the concentration of 33 mg mL^−1^. However, there was a change in the band of −OH and −NH, mainly in the width of band, being smaller, due to the lower concentration added and therefore lower presence of −OH groups. Therefore, the main interactions that occur between electrospun gelatin nanofiber and eggplant peel extract are hydrogen bonds of intermolecular hydrogen bonding type.

Kumar [40] mentions that when the −NH group of a peptide is involved in a hydrogen bond, its position changes, showing itself at a lower frequency, which may be the case in this study, when a decrease in amine band II is observed regarding control. Likewise, Li [41] performed a characterization of active gelatin-based films with the addition of natural antioxidants. They observed that the band related to the stretching of the N-H bands increased after the addition of the extracts, like the behavior obtained here. They attribute that the polyphenols present in the extracts contain several bands of −OH and C−O that form intra and intermolecular hydrogen bonds, as well as conjugated bonds. Jakobek [42] explains the process of the interaction of a protein with polyphenols, mentioning that these can be bonds by non-covalent hydrophobic interaction, and that can be stabilized by the hydrogen bond. This fact is because the −OH group of the phenolic compounds could be combined with the hydrogen acceptors of the gelatin molecule. In the same way, the paper mentions that the structure and molecular weight of the phenolic compounds is an important factor for the interaction, being able to observe in some studies that polyphenols with a high molecular weight can bind more strongly to proteins, so that the binding increases with the number of −OH groups present in the polyphenol molecule. Infrared nanospectroscopy gives us a better outlook for the structural and chemical analysis of materials, having been successfully applied in the evaluation of polymerization processes, characterizing their structure, surface, degradation, and modification of the polymer [43].

### 3.4. Encapsulation Efficiency

**Figure 6:**
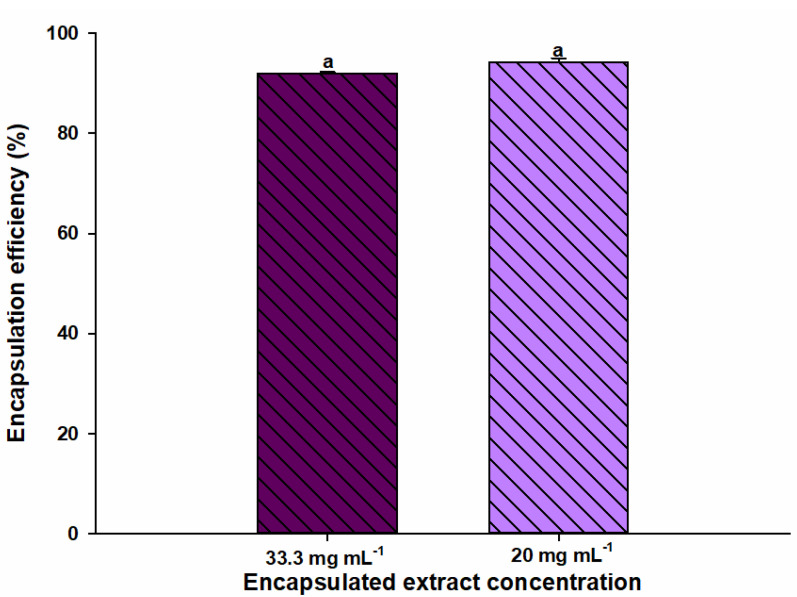
Encapsulation efficiency of eggplant peel extract-loaded electrospun gelatin nanofiber with 33.3 and 20 mg/mL of extract. Same letters in encapsulation efficiency represent no significant statistically differences by Tukey test (*p >* 0.05).

Figure 6 shows the EE of the nanoencapsules obtained in the present study. The EE is a very important parameter to be able to detect that the process was of quality or successful according to the encapsulated quantity. This is expressed in percent with respect to the total amount of extract used for nanoencapsulate. EE of eggplant peel extract-loaded electrospun gelatin nanofibers based on the phenolic compound quantification test was 92 and 94% for 33.3 and 20 mg mL^−1^, respectively. To have a process with high encapsulation efficiency, da Pereira et al. [44] described several key points, such as having a low solubility of the polymer to be used, high solubility of the solvent in water, high concentration of the polymer, having a high evaporation rate of the solvent and rapid solidification of the particles. Likewise, Horuz and Belibagli [17] performed a nanoencapsulation using zein obtaining results of more than 90% encapsulation, indicating very little loss of compounds using electrospinning. They also mention that the electrospinning technique has shown advantages compared to other nanoencapsulation techniques, since more favorable results have been observed in relation to EE. An example is spray drying where low values such as 38% have been reported for efficiency.

### 3.5. In Vitro Release of Eggplant Peel Extract

**Figure 7:**
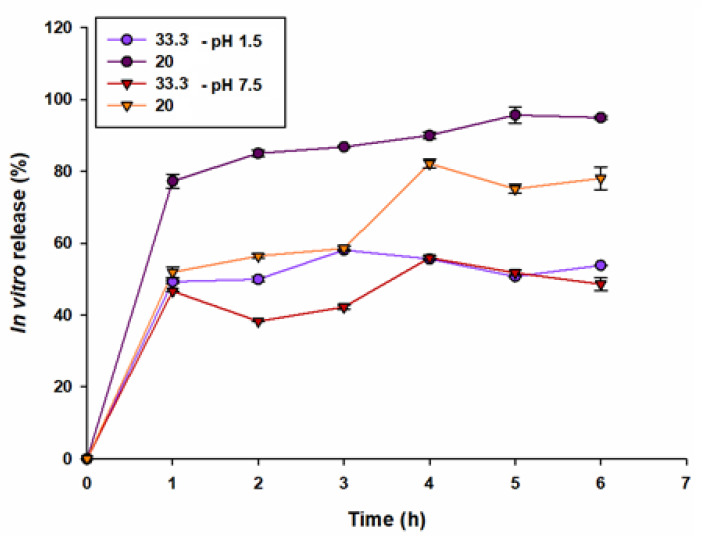
In vitro release at two pH (1.5 and 7.5) of eggplant peel extract-loaded electrospun gelatin nanofiber with 33.3 and 20 mg/mL of extract.

Figure 7 shows the in vitro release performed at two pH, 1.5 and 7.5. In order to achieve a successful release, several factors must be taken into account, such as the correct selection of membrane, chemical, nature, and temperature among others [45]. The result showed a maximum release of the encapsulated compounds of 95% for the treatment containing 20 mg mL^−1^ of the extract in the acid medium, leaving below the concentration of with 33.3%. The same behavior was observed in the neutral medium, where the maximum release was 82% and 52% for 20 and 33.3 mg mL^−1^, respectively, suggesting that it was an increase in upward release. Meng [46] mentioned that the best release of the compounds in shorter time in the medium of pH may be due to the reason that pH 1 is lower than the isoelectric point of the gelatin and the amino groups are protonized to carry positive charges, so the polymeric fibers with positive charges are rejected, achieving a flexibility of this, and being able to spread the content of the extract more easily. On the other hand, the behavior of pH 7 is due to the fact that, being near the isoelectric point of the gelatin, an almost zero solubility is presented.

## 4. Conclusions

Electrospinning using gelatin biopolymer is a good technique for the formation of electrospun nanofibers and encapsulating eggplant peel extract. Nanofiber morphology showed uniform and smooth fibers with the incorporation of the two concentrations of the extract, 33.3 and 20 mg mL^−1^. Furthermore, the extract addition increased fiber diameter; however, nanofibers with a tendency to monodispersity were obtained compared to the control nanofiber. FT-IR revealed conformational changes from gelatin powder into electrospun nanofiber and eggplant peel extract-loaded electrospun nanofiber; also, the main interactions formed were hydrogen bonds. This technique showed high encapsulation efficiency for two encapsulated extracts. The in vitro release of encapsulated compounds at a concentration of 20 mg mL^−1^ showed a maximum release time of 6 h, being almost 100%. Therefore, electrospun nanofibers have potential use in the food sector allowing the preservation of compounds and elaboration of nutraceutical products and functional foods.

Despite the favorable results, more studies must be carried out in vivo in food or murine models, as well as in vitro gastrointestinal systems to know with certainty the behavior of the release of the bioactive compounds present in the eggplant skin extract. With this, it can be applied in the food industry as a colorant in processed products with high pigment stability as additive, and in health, with greater bioavailability of compounds in the bloodstream and exerting a positive effect on the body.
